# Multi-Function Microelectromechanical Systems Implementation with an ASIC Compatible CMOS 0.18 μm Process

**DOI:** 10.3390/mi12030314

**Published:** 2021-03-17

**Authors:** Chih-Hsuan Lin, Chao-Hung Song, Kuei-Ann Wen

**Affiliations:** Department of Electronic Engineering, National Chiao Tung University, Hsinchu 300, Taiwan; nonox1257@gmail.com (C.-H.S.); stellawen@mail.nctu.edu.tw (K.-A.W.)

**Keywords:** microelectromechanical systems, three-axis magnetometer, three-axis accelerometer

## Abstract

A multi-function microelectromechanical system (MEMS) with a three-axis magnetometer (MAG) and three-axis accelerometer (ACC) function was implemented with an application-specific integrated circuit (ASIC)-compatible complementary metal-oxide-semiconductor (CMOS) 0.18 μm process. The readout circuit used the nested chopper, correlated double-sampling (CDS), noise reduction method; the frequency division multiplexing method; the time-division multiplexing method; and the calibration method. Sensing was performed by exciting the MEMS three-axis magnetometer at *X*/*Y*/*Z* axes mechanical resonant frequencies of 3.77/7.05/7.47 kHz, respectively. A modest die-level vacuum packaging resulted in in-plane and out-of-plane mechanical quality factors of 471–500 and 971–1000, respectively. The sensitivities of both the three-axis magnetometer with 2 mA driving current and the three-axis accelerometer were 7.1–10.7 uV/uT and 58.37–88.87 uV/ug. The resolutions of both the three-axis magnetometer with 2 mA driving current and three-axis accelerometer resolution were 44.06–87.46 nT/√Hz and 5.043–7.5 ng/√Hz. The resolution was limited by circuit noise equivalent acceleration (CNEM) and Brownian noise equivalent magnetic field (BNEM).

## 1. Introduction

In recent years, electronic devices, including microelectromechanical systems (MEMS) sensors, have occupied a very important position in the Internet of Things (IoT). The three-axis software gyroscope has attracted the attention of consumers. The magnetic sensor measures change in the three-axis geomagnetism with a mobile device and simulate the angular movement of the gyroscope through algorithms. As a result, three-axis heading information can be calculated.

Then, the magnetic sensor has the advantages of wide measurement range, high stability, no zero-temperature drift, low power consumption, low cost, anti-interference, and small size. Therefore, the calibration and diamagnetism of the magnetometer (MAG) are done well, and the MEMS gyroscope can be replaced by a three-axis MAG. To further reduce hardware cost, this paper proposes that a three-axis magnetometer and a three-axis accelerometer (ACC) share one microelectromechanical system (MEMS) structure.

Moreover, the readout circuit uses the nested chopper, correlated double-sampling (CDS) as well as other noise reduction technologies; the frequency division multiplexing method to read the three-axis ACC and three-axis MAG at the same time, and the time-division multiplexing method to reduce the mutual influence of each modulation frequency. Moreover, the calibration circuit corrects and compensates for the MEMS capacitance value that caused the variation during the process.

The design specifications are described as follows: To avoid increasing the complexity of the back-end readout circuit, when the minimum geomagnetic strength is 10 μT, the MAG can have a capacitance change of 2 aF to facilitate the effective elimination of the readout circuit noise amplification signal. The sensitivity of the MAG and ACC were 2 aF/μT and 200 aF/g. The sensing range of the MAG and ACC were 10–5000 μT and 1–10 g.

An ultra-sensitive Lorentz force MEMS MAG with a pico-tesla limit of detection is presented in Reference [1]. A single-structure three-axis Lorentz force MAG had a 30 nT/√HZ resolution in Reference [2]. The ACC readout circuit used a low-frequency noise reduction technique in Reference [3]. Development of a multi-axis complementary metal-oxide-semiconductor (CMOS)–MEMS resonant magnetic sensor Lorentz and electromagnetic forces were presented in Reference [4]. *Z*-axis MAGs for MEMS inertial measurement units using an industrial process are presented in Reference [5]. An elastic coefficient analysis on planar s-shaped micro-spring under high-impact load is presented in Reference [6]. An area-efficient three-axis MEMS Lorentz force MAG was the smallest Lorentz force sensor [7].

## 2. Materials and Methods

### 2.1. Process, Modeling, and Design of the MEM Sensor

The proposed MEMS ACC was fabricated using the United Microelectronics Corporation (UMC, Hsinchu, Taiwan) 0.18 μm 1-pol*Y*-6-metal (1P6M) standard CMOS–MEMS process provided by the Taiwan Semiconductor Research Institute (TSRI). This process can integrate the MEMS structure with the CMOS readout circuit in a single chip without any post-processing after wafer out. The post-CMOS fabrication was performed using a dry etching process.

The typical differential equation of mechanical equilibrium is shown in Reference [8] and takes Laplace transform and replaces the s term with jω, the frequency response of displacement to external actuation force, quality factor Q, natural frequency ω_0_, and Hooke’s law is given as:

(1) $$\frac{x\left(s\right)}{F_{\mathrm{ext}}\left(s\right)}=\frac{k_{e}{}^{-1}}{\left(1-\frac{w^{2}}{w_{0}^{2}}\right)+\frac{\mathrm{jw}}{{\mathrm{Qw}}_{0}}}\;;\;Q\;=\;\;\frac{\sqrt{m_{e\;}k_{e}}}{b_{e}}\;;\;w_{0}=\sqrt{\frac{k_{e}}{m_{e\;}}};\;F_{\mathrm{ext}}=k_{e}x\;\mathrm{if}\;w<<w_{0}$$

where an effective mass m_e_ and its displacement is x, an effective spring constant k_e_, and an effective damping coefficient b_e_, any external actuation force $F_{\mathrm{ext}}$.

### 2.2. Structure of the MEMS Magnetometer

**Figure 1:**
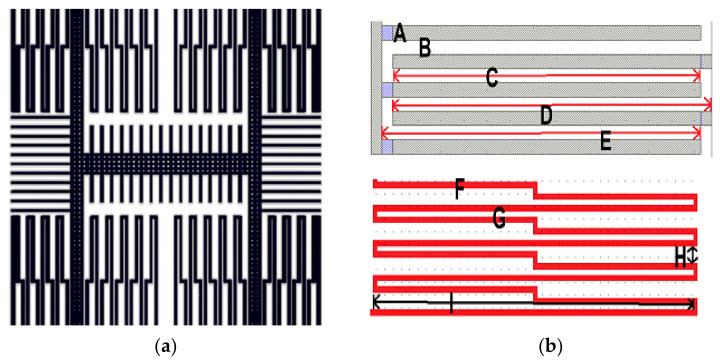
(**a**) The layout of the microelectromechanical system (MEMS) magnetometer; (**b**) marks on the combs and springs of the structure.

Figure 1a illustrates the layout and model of the MEMS MAG. The structure was composed of a stator, rotor, anchors, fingers, springs, and proof mass. The stator is the part that is not released in the CMOS–MEMS process, and the rotor is the part that can be actuated—anchors fix the structure—and provide the input or output of the Lorentz currents, while fingers are sensing the variation in the capacitance.

Considering the influence of different spring structures on the displacement of the sensor, the elastic coefficient of the S-shaped spring structure is inversely proportional to the third power of the length, and the coefficient is relatively small, which can effectively make the sensor produce displacement [7]. In this paper, the length and the number of folding springs as the S-shaped spring structure decided the spring constant and further affected the resonance frequency as shown in Figure 1b. In addition, the length of the beam will influence the curvature of the structure. The dimension of the structure is shown in Table 1 and A–I are the dimensions of the structure’s different parts and are marked in Figure 1b and Table 1.

### 2.3. Operational Principle of the MEMS Magnetometer

The magnetic field $\vec{B}\left(j\omega \right)$ with the applied current $\vec{I}\left(j\omega \right)$ flowing through the suspended conductor of length L, results in the Lorentz force $\vec{F_{\mathrm{mag}}}\left(j\omega \right)$, which can be shown as $\vec{F_{\mathrm{mag}}}\left(j\omega \right)=\mathrm{LI}\left(\vec{l}\left(j\omega \right)\times \vec{B}\left(j\omega \right)\right)$ [8].

**Figure 2:**
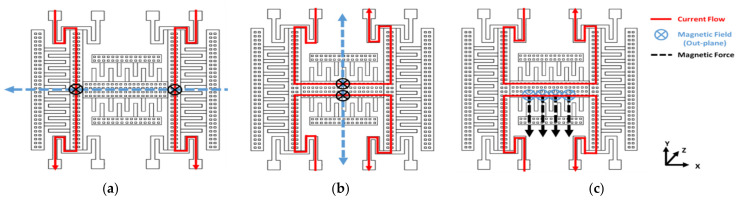
(**a**) Schematic of the sensing *X*-axis magnetic field; (**b**) schematic of the sensing *Y*-axis magnetic field; (**c**) schematic of the sensing *Z*-axis magnetic field.

To simplify the analysis of $\vec{F_{\mathrm{mag}}}\left(j\omega \right)$ and supposing the frequency is much less than the resonance frequency, the frequency of $\vec{F_{\mathrm{mag}}}\left(j\omega \right)$ is determined mainly on the frequency of the Lorentz current $\vec{I}\left(j\omega \right)$. When the Lorentz current flows through the MEMS, the Lorentz force will be generated perpendicular to the external magnetic field and detect three-axial magnetic fields as are shown in Figure 2a–c.

## 3. The Finite Element Method (FEM) Simulation Result of the MEMS Sensor

**Figure 3:**
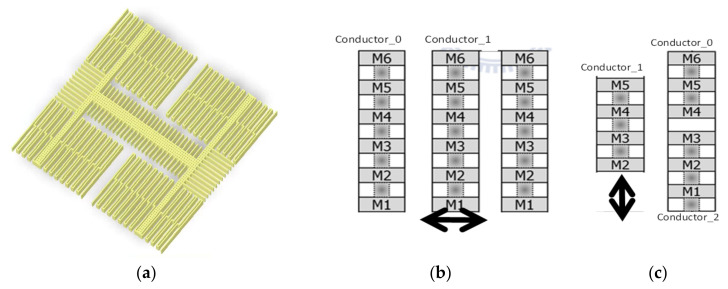
(**a**) The 3D model of the MEMS sensor created by CoventorWare; (**b**) capacitor for detecting magnetic field in the *Z*-direction; (**c**) capacitor for detecting magnetic field in *X*- and *Y*-directions.

First, based on the UMC CMOS–MEMS process, we drew the layout of the structure in the virtuoso tool to extract the layout file and import it into the mechanical analyzer for CoventorWare tool, to build a 3D model (Figure 3a). In the simulation, the dimensions of the element unit were selected to be $0.5\times 0.5\times 0.5{\;\mu m}^{3}$.

First, this study used the CoventorWare tool to simulate the initial sensing capacitance value and assume that the curvature of the structure was caused by the process to make the overlap part of the finger 80% of the original. When detecting the *Z*-axis magnetic field, the sensor moved in the plane direction, the capacitive comb was stacked METAL 1 – METAL 5 as both the conductor_0 (stator) and conductor_1 (rotor), shown in Figure 3b, and the simulation result was $C_{0}\;$= 4.53×0.8×40=144.96 fF. The number of the finger was 40.

When detecting the *X*- or *Y*-axis magnetic field, the sensor moved out of the plane, the capacitive comb was stacked METAL 2~5 as conductor_1 (the rotor), and both conductor_0 with METAL 1~3 and conductor_2 with METAL 4~5 as the stator. The average of the initial capacitance value simulation results were $C_{0}=\left[\frac{1.512+1.494}{2}\right]\times 0.8\times N=10.8,\;21.6$ fF and is shown in Figure 3c. The number of N was 9, and 18 corresponded to the X/Y axes.

Since CoventorWare cannot directly simulate the size of the magnetic field, according to the Lorentz force formula, F = L*I*B, the applied magnetic force was converted to the pressure that CoventorWare can simulate; thus, the expected applied current was 2 mA [9,10]. According to the formula, the length of the line converted the magnetic field of 0–5000 μT into the pressure applied to the beam to simulate the deformation of the structure. Then, we could simulate the deformation of the structure when the *X*- and *Y*- axes were subjected to a magnetic field of 0–5000 μT with an interval of 200 μT. The simulation result shows that the displacement was 0.0256 μm and 0.01 um when subjected to 5000 μT. The sensitivity was approximately $5.12\times 10^{-6}\;\mathrm{and}\;2\times 10^{-6}\;\frac{\mu m}{\mu T}$.

Besides, when the MAG was subjected to a magnetic field of 5000 μT in the *Z*-axis direction, the displacement distribution in the *Y*-direction, and the maximum displacement was approximately 0.0045 μm, and the gap width was 2.6 μm. It confirms the high linearity of the displacement into the capacitance difference and the sensitivity was approximately $9\times 10^{-7}\;\frac{\mu m}{\mu T}$.

**Figure 4:**
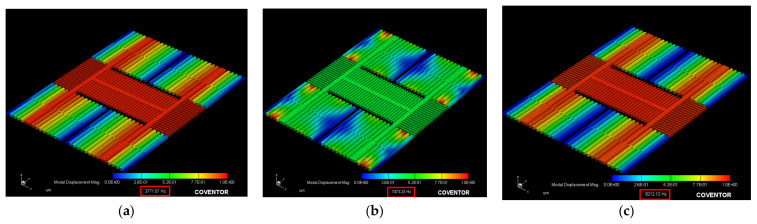
Modal analysis for the accelerometer movement along the (**a**) *X*-, (**b**) *Y*-, and (**c**) *Z*-axes.

Then, the resonance frequency, $f_{O}$, the simulation was completed by the modal analysis of the MemMech module of CoventorWare tool. The proper boundary conditions were then set in the setup dialog of the solver. The *X*/*Y*/*Z* axes resonance frequencies were 3.77/7.05/7.47 kHz, and the *X*/*Y*/*Z* axes generalized masses were 16.12/5.8/5.12$\;\times 10^{-10}\;\mathrm{kg}$ and generated 3D images shown in Figure 4a–c.

**Figure 5:**
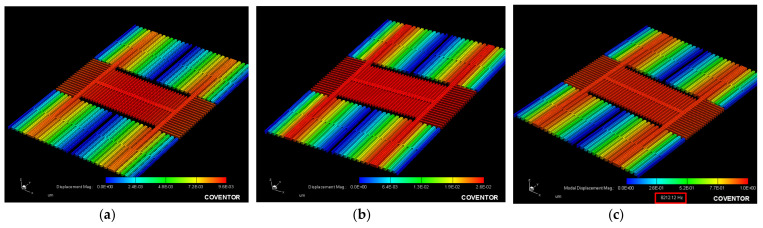
The magnetometer shows the plane motion direction along the (**a**) *X*-, (**b**) *Y*-, and (**c**) *Z*-axes.

By the shift of the *X*/*Y*/*Z* axes modal analysis, we can ensure the direction of the design current and make the structure move in the correct corresponding modal. The MAG’s plane motion direction along the *X*/*Y*/*Z* axes are shown in Figure 5a–c.

Because the Q value has an important influence on the structure, we used CoventorWare to simulate the damping constant. The general damping constant was divided into squeezed and slide, and can be expressed as Formula (2) [6].

(2) $$b_{\mathrm{squeeze}}=7.2\times N_{\mathrm{gap}}\times {\mu l}_{\mathrm{ov}}\times \frac{t^{3}}{d^{3}}\;;{\;b}_{\mathrm{slide}}=N_{\mathrm{gap}}\times \mu \times \frac{A}{d}$$

N_gap_ is the number of intervals, μ is the viscosity coefficient of air, it was approximately $1.86\times 10^{-5}\;\frac{\mathrm{kg}}{\mathrm{ms}}$ at room temperature, $l_{\mathrm{ov}}$ is the overlapping length of the capacitor plates, t is the thickness, d is the distance between the stator and the rotor, and A is the area where the capacitor plates overlap.

Then the Knudsen number (Kn) was added to modify the viscosity coefficient in consideration of the interaction between air molecules and structural systems. Because the free path of air molecules is about 100 um in a low-pressure environment and the plate spacing of this structure was 2.6 um, $K_{n}≅\frac{100}{2.6}=38.46$ could be obtained. The formula for modifying the viscosity coefficient is given as (3):

(3) $$\mu_{\mathrm{eff}}=\frac{\mu }{1+9.638K_{n}{}^{1.159}}=2.8\times 10^{-8}\;\frac{\mathrm{kg}}{\mathrm{ms}}$$

By calculating the above Formula (2), the damping constant could be calculated and is given in (3). It can be seen from Formula (4) and $b_{\mathrm{squeeze}}$ that it almost dominates the damping coefficient:

(4) $$b_{\mathrm{squeeze}}=7.2\times N_{\mathrm{gap}}\times \mu_{\mathrm{eff}}l_{\mathrm{ov}}\times \frac{t^{3}}{d^{3}}≅8.1\times 10^{-8}\;\frac{\mathrm{kg}}{s}\;;{\;b}_{\mathrm{slide}}=N_{\mathrm{gap}}\times \mu_{\mathrm{eff}}\times \frac{A}{d}≅7.61\times 10^{-10}\;\frac{\mathrm{kg}}{s}$$

Then, the Q value can be estimated as $Q=\frac{\sqrt{m_{e\;}k_{e}}}{b_{e}}=\frac{2{\pi f}_{0}M}{b}$, $f_{0}$ is the resonance frequency, M is the mass, $Q_{x}$ is 471–500, and $Q_{z}$ is 971–1000.

The displacement and the distribution of the finger number N were obtained and by $\Delta C≅C_{0}\times \frac{\Delta d}{d}$. The capacitance change corresponding to the applied 200 μT magnetic fields could be calculated by $\Delta C_{x}=3.402\;\mathrm{aF}$, and the movement at the *Z*-axis resonance frequency was approximately $\Delta C_{x}=3.402\;\mathrm{aF}\times 1000=3.402\;\mathrm{fF}$, $\Delta C_{y}=2.659\;\mathrm{aF}$, and the movement at the *Z*-axis resonance frequency was approximately $\Delta C_{y}=2659\;\mathrm{aF}\times 1000=2.659\;\mathrm{fF}$ and $\Delta C_{z}=8.02\;\mathrm{aF}$, and the movement at the *Z*-axis resonance frequency was approximately $\Delta C_{z}=8.02\times 500=4.01\;\mathrm{fF}$.

**Figure 6:**
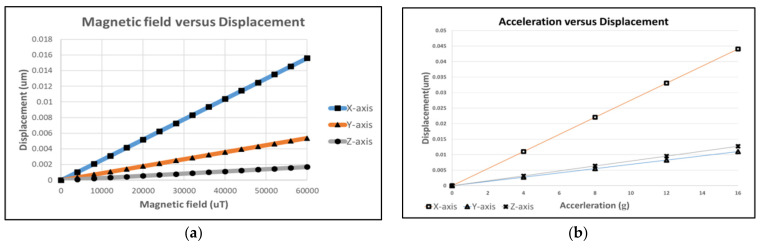
(**a**) Acceleration versus displacement of the three-axis; (**b**) magnetic field versus displacement of the three-axis.

In addition, the displacements of the ACC at the *X*/*Y*/*Z* axes are shown in Figure 6a for 2–10 g acceleration, and the displacements of the MAG at the *X*/*Y*/*Z* axes are shown in Figure 6b for 0–60,000 μT magnetic field. Then, we calculated the three-axis capacitance change value for 2 g acceleration,$\;\Delta C_{x}=0.05543\times \mathrm{Nx},$ $\Delta C_{y}=0.0093\times \mathrm{Ny},$ and $\Delta C_{z}=0.0039\times \mathrm{Nz}$; Nx/Ny/Nz is a three-axis finger number. When Nx/Ny/Nz was 6/34/56, the *X*/*Y*/*Z* axes sensitivities of the MAG were 17.01/13.3/20.05 aF/μT. The *X*/*Y*/*Z* axes sensitivities of the ACC were 166.5/159/109.35 aF/g.

Finally, the equivalent Brownian noise (BNEA) was calculated, where $k_{B}$ is the Boltzmann constant and equal to $1.381\times 10^{-23}\;J\Delta K^{-1}$, T is the absolute temperature, m is the mass of proof mass, and b is the damper coefficient [6,11]:

(5) $$\mathrm{BNEA}=\frac{\sqrt{4k_{b}\mathrm{Tb}}}{9.8m}\left(\frac{g}{\sqrt{\mathrm{Hz}}}\right)≅1.44615\;\frac{\mu g}{\sqrt{\mathrm{Hz}}}$$

The Brownian noise equivalent magnetic field (BNEM) was calculated, where L is the effective current length, i is current, Q is the quality factor of the MEMS structure, k is the spring constant, and $f_{O}$ is the resonance frequency [12].

(6) $$\mathrm{BNEM}=\frac{\sqrt{4k_{b}\mathrm{Tk}/2{\pi f}_{O}Q}}{\mathrm{Li}}\;\frac{T}{\sqrt{\mathrm{Hz}}}$$

This study used CoventorWare to simulate the applied pressure and convert it into force and displacement, and then we calculated the equivalent spring constant according to Hooke’s law and F = kx. When the MAG is operating, it will only vibrate in the *X*- and *Z*-directions. The Calculated X and *Z*-axis spring constant is shown in (7).

The results of the spring constant calculation show that the *Z*-axis was relatively difficult to vibrate and the equivalent spring was harder, which is in line with the large resonance frequency in the previous simulation and the process limitation in the *Z*-axis direction. The BNEMs of the *X*/*Y*/*Z* axes are shown in (8)–(10).

(7) $$k_{x}=\frac{7.706421E-03}{8.993802E-03}=0.857\;\frac{N}{m};\;k_{z}=\frac{1.03688E-01}{2.563647E-02}=4.0446\;\frac{N}{m}$$

(8) $${\mathrm{BNEM}}_{x}=\frac{\sqrt{4k_{b}\mathrm{Tk}/2{\pi f}_{n}Q}}{\mathrm{Li}}\;\frac{T}{\sqrt{\mathrm{Hz}}}=\frac{5.566*10^{-14}}{642.2*2*10^{-9}}=43.33\;\frac{\mathrm{nT}}{\sqrt{\mathrm{Hz}}}$$

(9) $${\mathrm{BNEM}}_{y}=\frac{3.777*10^{-14}}{299*2*10^{-9}}=63.16\;\frac{\mathrm{nT}}{\sqrt{\mathrm{Hz}}}$$

(10) $${\mathrm{BNEM}}_{z}=\frac{2.345*10^{-14}}{642.2*2*10^{-9}}=18.26\;\frac{\mathrm{nT}}{\sqrt{\mathrm{Hz}}}$$

Moreover, when the MEMS structure is to be used as a MAG, a bias current must be applied to the structure to generate a Lorentz force.

## 4. Circuit Design and Simulation

### 4.1. System Architecture

**Figure 7:**
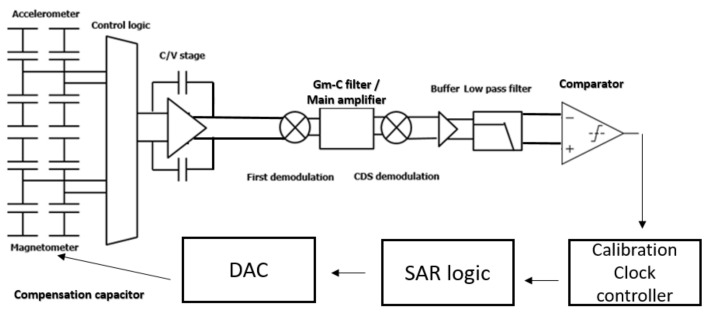
The overall architecture of the proposed readout system. Capacitance-to-voltage (C/V) circuit; successive approximation register (SAR) logic; digital-to-analog converter (DAC); the operational transconductance amplifier-capacitor (GM-C) filter.

The system architecture is shown in Figure 7. It can be divided into a readout circuit and the calibration circuit. The variation in the capacitance from the MAG/ACC was modulated with a 400 kHz pulse signal converted into a voltage signal by a capacitance-to-voltage (C/V) circuit. The readout circuit included the nested chopper amplifier, C/V stage, first demodulation, the operational transconductance amplifier-capacitor (GM-C) filter, main amplifier, CDS demodulation, buffer, the two cascading resistor-capacitor (RC) filters, the frequency division multiplexing circuit, and the time-division multiplexing circuit. The calibration circuit included the comparator, calibration clock controller, successive approximation register (SAR) logic, and digital-to-analog converter (DAC).

### 4.2. Readout Circuit

The nested chopper amplifier was implemented to reduce the residual offset of a chopper amplifier [13]. For example, the higher modulation frequency was 400 kHz and the lower modulation frequency was 25 kHz, so the theoretically achievable improvement in residual offset was the ratio of 400 kHz and 25 kHz, which means the residual ratio would be 16 times less compared to the conventional chopper amplifier.

**Figure 8:**
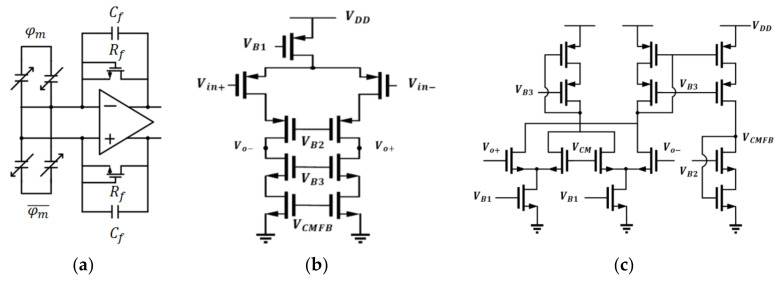
The schematic of the (**a**) capacitance-to-voltage (C/V) stage; (**b**) telescopic operational amplifier; (**c**) the schematic of continuous-time common-mode feedback (CMFB) circuit.

The first stage of the readout circuit was the C/V stage used to convert the variation in the capacitance into a voltage signal. The circuit is shown in Figure 8a–c. The large feedback resistor, $R_{f}$, was implemented by a sub-threshold n-type metal-oxide-semiconductor (MOS) to bias the DC voltage of input ports to be half of the supply voltage and had much less area than a poly resistor, small leakage current. Since the output range at this stage is quite small, which is approximately 10 mV, telescopic topology was implemented and the gain was 76.46/77.81/73.62 dB, the phase margins (PMs) were 61.2/61.8/61.2 degrees, gain–bandwidths (GBW) were 22.06/22.31/21.32 MHz for a typical-typical (TT)/slow-slow (SS)/fast-fast (FF) corner. In addition, for a fully differential operational amplifier, continuous-time common-mode feedback (CMFB) circuit was designed and the gains were 76.54/77.91/75.39 dB, the PMs were 75.41/74.79/75.57 degrees, and the GBWs were 3/3.2/29 MHz for a TT/SS/FF corner.

The input-referred noise of the first stage dominates the overall noise of the readout circuit, so the operational amplifier was designed to have a large size p-type MOS input pair to decrease the flicker noise. Similarly, output transistors with large overdrive voltage were chosen to reduce the thermal noise. The noise contributions at 250 Hz were 210.37/206.52/206.42 nV/$\sqrt{\mathrm{Hz}}$ for a TT/SS/FF corner.

After analyzing the amplifier, from Figure 8a, the transfer function of the C/V stage can approximately be expressed as:

(11) $$V_{o+}-V_{o-}≅\left(\varphi_{m}-\bar{\varphi_{m}}\right)\times \frac{2\Delta C}{C_{f}\left(1+\frac{1}{{\mathrm{SC}}_{f}R_{f}}\right)}$$

where ΔC, $C_{f}$, $\varphi_{m}$, and $R_{f}$ are the variation of the MEMS capacitance, feedback capacitor, modulation clock, and effective resistance of MOS, respectively. The amplification gain was determined by the ratio of the variation in the capacitance and feedback capacitor.

Then, the capacitive sensing scheme of the fully differential bridge was as follows. The variation in the capacitance would induce a charge transition and generate an A/C voltage, and a capacitive divider would provide the voltage–domain scheme of the sensing process, where $C_{0}$ is the initial capacitance between stator and rotor of the MEMS, ΔC is the variation in the capacitance with a magnitude equal to $\left|C_{0}\times \frac{d}{d-d_{0}}\right|$, $d_{0}$ is the gap spacing between fingers and d is the displacement caused by the natural actuation, $C_{p}$ is the parasitic capacitance at the sensor output node, and $V_{m}$ is the full-scale of the modulation pulse signal. The sensed voltage is given by [6]:

(12) $$V_{\mathrm{sense}}=V_{p}-V_{n}=2\times \frac{2C_{0}}{2C_{0}-C_{p}}\times \frac{d}{d_{0}}\times V_{m}$$

**Figure 9:**
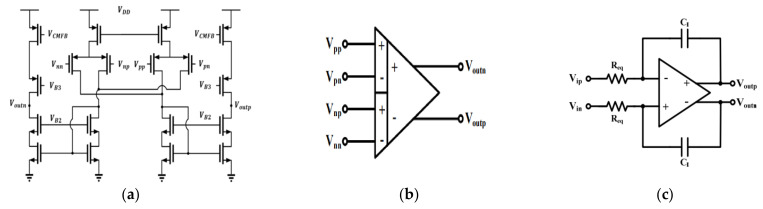
(**a**) The schematic of differential-difference amplifier (DDA) circuit; (**b**) the symbol of a DDA circuit; (**c**) full*Y*-differential Miller integrator.

The second stage is the amplification stage used to amplify the signal in the range of tens of millivolts to a large range. The band-pass amplifier was implemented to amplify the modulated signal in the high-frequency band and reduce low-frequency noise from the first stage. The band-pass filter was composed of a differential-difference amplifier (DDA) and a Miller integrator as shown in Figure 9a–c.

The transfer function of the band-pass amplifier is:

(13) $$T\left(s\right)=\frac{V_{\mathrm{out}}\left(s\right)}{V_{\mathrm{in}}\left(s\right)}=\frac{G\left(s\right)}{1-G\left(s\right)H\left(s\right)}=\frac{-{s\tau A}_{0}}{s\tau +A_{0}}=\frac{-{\mathrm{sR}}_{\mathrm{eq}}C_{I}A_{0}}{{\mathrm{sR}}_{\mathrm{eq}}C_{I}+A_{0}}$$

where $R_{\mathrm{eq}},{\;C}_{I},{\;A}_{0}$ are the equivalent resistances of the integrator, integrating capacitor, and low frequency (D/C) gain of the DDA. A transfer function is a typical form of a band-pass amplifier. Figure 9c shows the DDA and the schematic of the Miller integrator.

The transfer function of DDA is:

(14) $$V_{\mathrm{outp}}-V_{\mathrm{outn}}=A\times \left[\left(V_{\mathrm{PP}}-V_{\mathrm{PN}}\right)-\left(V_{\mathrm{NP}}-V_{\mathrm{NN}}\right)\right]$$

From Formula (13), the time constant, τ, the high-pass cutoff frequency is defined by $R_{\mathrm{eq}}$ and $C_{I}$. The gains at 100 kHz were 14.57/14.43/14.75 dB for a TT/SS/FF corner and at 100 Hz were −8.74/−7.93/−10.86 dB for a TT/SS/FF corner, and the gain rejections were 23.31/22.36/25.61 dB for a TT/SS/FF corner between the signal band and undesired band.

**Figure 10:**
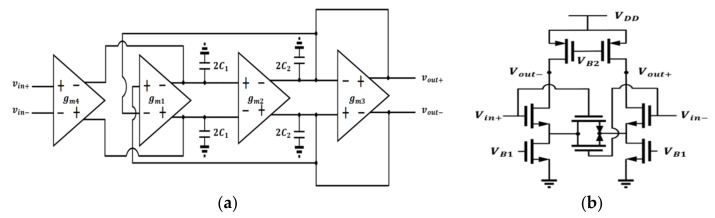
(**a**) The second order bi-quad GM-C filter; (**b**) schematic of the Gm cell unit.

After the first demodulation, the signal band was converted to approximately 25 kHz. Hence, the second-order bi-quad filter with a GM-C, shown in Figure 10a, was used to separate the demodulated signal and undesired signal.

By connecting gm cells as shown in Figure 10a, a second-order transfer function, the natural frequency $\omega_{0}$, and the Q factor can be achieved which is equal to (15).

(15) $$\frac{v_{\mathrm{out}}}{v_{\mathrm{in}}}=\frac{g_{m2}\times {\mathrm{gm}}_{4}}{C_{1}C_{2}s^{2}+g_{m3}C_{1}s+g_{m1}g_{m2}};\;\omega_{0}=\sqrt{\frac{g_{m1}g_{m2}}{C_{1}C_{2}}};\;Q=\frac{1}{g_{m3}}\times \sqrt{\frac{g_{m1}g_{m2}C_{1}}{C_{2}}}$$

Thus, by controlling the transconductance of cells and capacitors, the cutoff frequency and Q factor can be determined. The gains were −0.18/−0.17/−0.19 dB, 3 dB bandwidths were 25.62/26.54/24.96 kHz for a TT/SS/FF corner.

However, the most common problem of the GM-C filter is the linearity of the Gm-cell, because the transconductance value of the input transistor will dramatically vary as the input voltage changes. To maintain the linearity of the filter, the Gm-cell with a source degeneration, shown in Figure 10b, was implemented. Thus, with the additional transistor serving as a resistor, the effective transconductance of the Gm cell was nearly a fixed value with input signal changes from 0.7–1.1 V.

The CDS circuit is combined with the demodulation function [6]. The CDS stores the flicker noise and DC offset in the capacitor in two phases. In phase one, the CDS samples the differential signals. In the next phase, the differential signals and error voltage will be subtracted. As a result, the noise far below the switching frequency and DC offset will be canceled and the amplitude of the signal will be twice as large.

Because there is a large capacitor between the filter and the CDS circuit, the CDS circuit cannot provide such a high current to push the capacitor. There should be a buffer after the CDS demodulator. The buffer’s output range under a TT/SS/FF corner was 0.7–1.1 V and met the specifications of the output range, which were 0.5–1.2 V [14].

**Figure 11:**
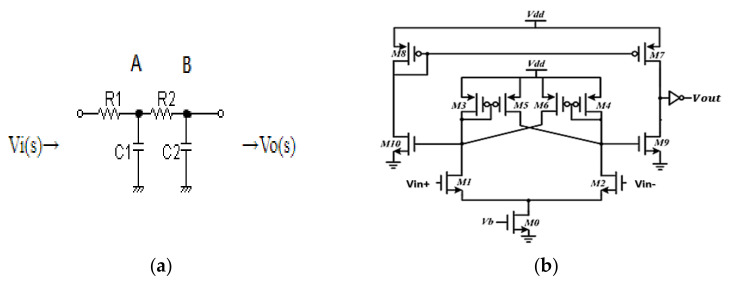
(**a**) The two cascading resistor-capacitor (RC) filters; (**b**) the continuous-time comparator circuit.

The topology of an active, fully differential filter requires the use of a CMFB circuit, which constraints output swing due to the headroom voltage of the transistor in the CMFB circuit. Thus, the RC filter was implemented for the filtering function, and the two cascading RC filters are shown in Figure 11a.

### 4.3. The Calibration Circuit

From Figure 11a, the two cascading filters have two nodes: A and B. We had a larger bandwidth in node A, which meant that the settling time of the calibration operation could decrease well as soon as we set the value of $R_{1}$ and $C_{1}$ quite smaller than $R_{2}$ and $C_{2}$. The settling time in node A was much shorter than node B when the pulse width was 160 us; node B served as the output node.

**Figure 12:**
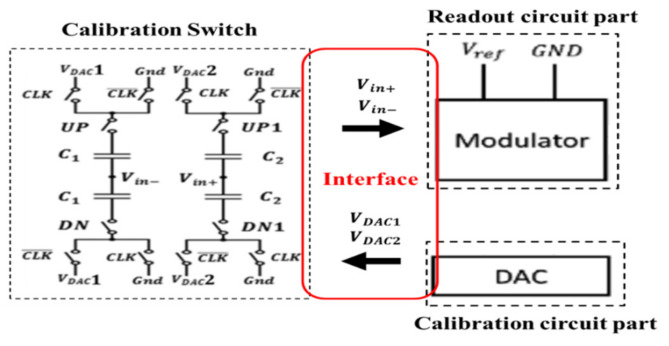
Calibration switches and interface of the readout and calibration circuit. The UP/DN are the switches in left side; The UP1/DN1 are the switches in right side.

In the calibration circuit, the continuous-time comparator is shown in Figure 11b and was used to determine the polarity of the offset. The current-mirror pre-amplifier followed by a positive feedback latch was used. After comparing the D/C voltage at node A of the two cascading filters, the control logic circuit controlled the UP or DN/UP1 or DN1 switches to reduce the DC offset in differential sides in Figure 12.

The offset of the comparator was the important specification and compared a 500 Hz sinusoidal wave and a reference voltage to check the offset. The Monte Carlos simulation included the global process variation and mismatch. Under sigma and iteration equal to three and 100, the offset was varied from −4.8–4 mV for TT/SS/FF corners. Compared to the resolution in the DAC, which was equal to $\frac{900\mathrm{mV}}{32}=28.125\left(\mathrm{mV}\right)$, it would not affect the correction of the logic circuit.

In the chopper amplifier, there are switches in the interface of calibration and readout circuit. Thus, we can compensate the unbalanced capacitance by C1 and C2 between $V_{\mathrm{in}+}$ and $V_{\mathrm{in}-}$ nodes. The calibration switch is suitable for the chopper amplifier as shown in Figure 12 [15]. The calibration switch can reduce the DC deviation between $V_{\mathrm{in}-}{\;\mathrm{and}\;V}_{\mathrm{in}+}$ by controlling the $V_{\mathrm{DAC}1}$/$V_{\mathrm{DAC}2}$ and the UP or DN/UP1 or DN1 switches for coarse/fine calibration operations. This paper proposes to directly use the SAR logic to approach and eliminate the deviation value. This method can save unnecessary approach time with a low-pass filter and can achieve faster (80%) than that in Reference [15] correction time under the same bit number.

The $C_{1}$ of the coarse calibration operation was designed to be 32 times larger than the $C_{2}$ of the fine calibration operation to obtain 5 bits resolution. The dimension of the unit capacitance was 1 μm×1 μm and the value was 1 fF. The resolution of the calibration capacitance was evaluated as $\frac{1\left(\mathrm{fF}\right)}{2^{5}}\times \frac{0.6}{0.9}≅20.83\left(\mathrm{aF}\right)$, $2^{5}$ came from 5 bit DAC and $\frac{0.6}{1.8}$ was because the full scale of $V_{\mathrm{DAC}1}$ and $V_{\mathrm{DAC}2}$ was 0.6 V and $V_{\mathrm{REF}}$ was 0.9 V. Moreover, the maximum calibration capacitance was $\left(\frac{32}{1.5}\right)≅21.33\left(\mathrm{fF}\right)$.

**Figure 13:**
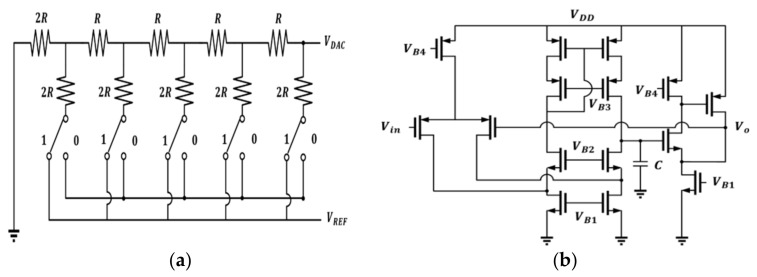
(**a**) The resistor-2resistor ladder (R-2R) digital-to-analog (DAC) circuit; (**b**) schematic of the unit gain voltage follower. $V_{\mathrm{DAC}}$, the voltage of digital-to-analog converter; $V_{\mathrm{REF}}$, reference voltage; $V_{\mathrm{in}}$, the input signal voltage; $V_{O}$, the output voltage.

A 5-bit resistor-2resistor ladder digital to analog (R-2R DAC) circuit is shown in Figure 13a and has little influence on integral nonlinearity (INL) and differential nonlinearity (DNL). In addition, the additional resolution can be obtained by setting two sets of switches and smaller calibration capacitors, $C_{1},{\;C}_{2}$, in Figure 12 as well. Thus, a 5 bit DAC is enough in this design. In this design, the unit resistance is 1 kΩ and R is 4 kΩ to get a proper trade-off between the layout area, deviation caused by the process, and power consumption. When the digital code was 5′b0000 ~ 5′b1111, the analog output was equal to $V_{\mathrm{REF}}=0-0.6\;V$.

Since the output of the DAC circuit was designed to connect to the control switch, which had a small conductance resistance, if we directly connected $V_{\mathrm{DAC}}$ to switches, the value of $V_{\mathrm{DAC}}$ would be critically affected by the finite resistance. As a result, the unit gain buffer must provide infinite input impedance and small output impedance. The schematic of the voltage follower composed of a folded-cascode and a super source follower is shown in Figure 13b. The folded-cascode with PMOS-type input pair was chosen because the input range can be nearly close to 0 V. A super source follower was designed to have a small output impedance to drive the resistive control switch. We input a pulse signal from 0 to 0.9 V and the following range is from 4.646 to 800 mV. It resulted in an error that came from misjudging 0 V as 4.646 mV and can be evaluated as $\frac{1\left(\mathrm{fF}\right)}{2^{5}}\times \frac{4.646\left(\mathrm{mV}\right)}{1.8\left(V\right)}≅0.08\;\left(\mathrm{aF}\right)$.

**Figure 14:**
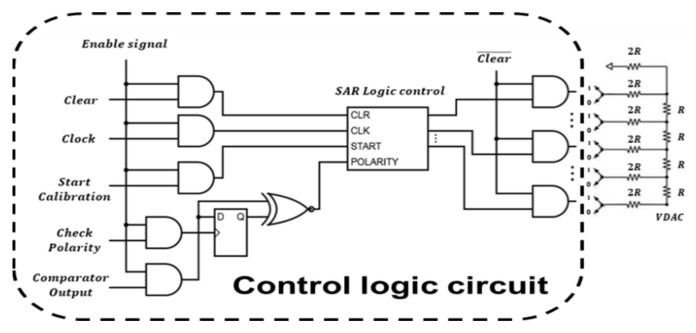
Calibration logic circuit.

The operation of the calibration was based on the control logic circuit in Figure 14. When the enabled signal was pulled high, the polarity of the output of the comparator would be sent to the input (D) of the D-flip-flop. Then, checking if the polarity was high, the SAR logic circuit would start to generate the digital output to the DAC circuit.

**Figure 15:**
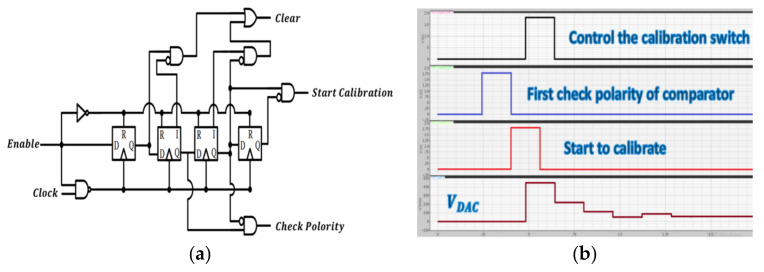
(**a**) Logic gate diagram of the control signal generator; (**b**) transient simulation of the control signal circuit. Enable, the enable signal; Clock, the clock signal; Start Calibration, output the calibration signal; Check Polarity, output the polarity of the comparator.

**Figure 16:**
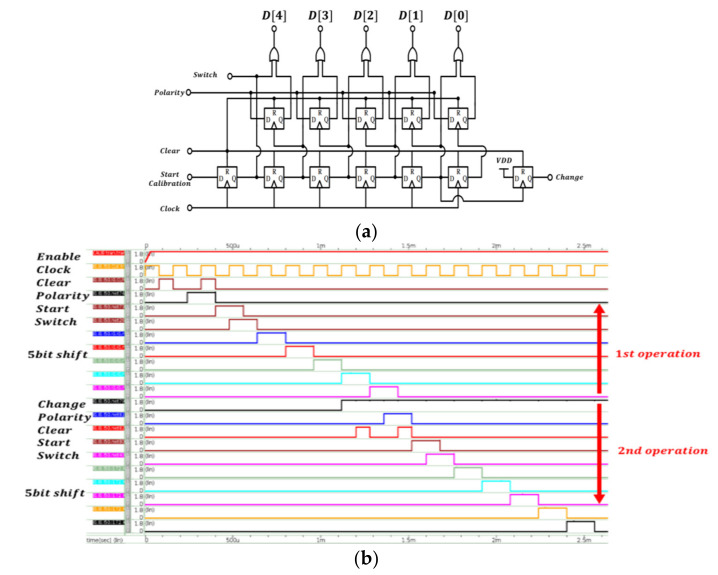
(**a**) Proposed SAR logic circuit; (**b**) the operational sequence of SAR logic circuit. Clear, the reset signal; Start Calibration, start to the calibration signal; Polarity, from the polarity of the comparator; Clock, the clock signal; Switch, output to control the calibration switch; Change, divide the first operation and the second operation.

The control signal generator is shown in Figure 15a and controls the proposed SAR logic circuit shown in Figure 16a. The circuit triggered by the clock generates the control signal along the positive edge of the clock step by step to achieve correct logic functions. The simulation results are shown in Figure 15b. First, the calibration circuit is enabled, and the polarity is checked. Then, the calibration circuit starts and controls the switch (UP, DN/UP1, or DN1), and the output of DAC starts to change the value of the analog output corresponding to the difference in the DC offset.

Finally, the SAR logic circuit is illustrated in Figure 16a. Then, as D(0), D(1), D(2), D(3), and D(4) are all output, the “Change” signal will be pulled high to trigger the second operation, which is a fine calibration operation and triggers the second SAR circuit to work. After the first operation and second operation, the work of the calibration circuit is done. The operational sequence of the SAR logic circuit is shown in Figure 16b.

### 4.4. Co-Simulation With MEMS, Readout Circuit, and the Calibration Circuit

The equivalent capacitance can be described as a four-ports Verilog-A model, which is given by:

(16) $$C_{c}=C_{i}+\Delta C\;\times \left(V\left(V_{\mathrm{in}},V_{\mathrm{sub}}\right)\right)\;;\;I_{c}\left(V_{p},V_{n}\right)<+\mathrm{ddt}\left(\Delta C\times V\left(V_{p},V_{n}\right)\right)$$

where the total capacitance of MEMS capacitance C_c_ is equal to the summation of initial C_i_ and variant capacitance ∆C. Both V_in_ and V_sub_ are two ports given a sinusoidal wave with a frequency that is the frequency of the input acceleration and Lorentz current to drive the model. In this design, the frequency of acceleration was set to be 100 Hz, and both V_p_ and V_n_ were the voltage of the top and bottom of the MEMS capacitive model. The C_i_ and ∆C of both three-axis ACC in the Verilog-A model were 21/123/67.33 fF and 166.5/159/109.35 aF. The C_i_ and ∆C of both three-axis MAG in the Verilog-A model were 10.8/21.6/144.96 fF and 17.01/13.3/20.05 aF.

**Figure 17:**
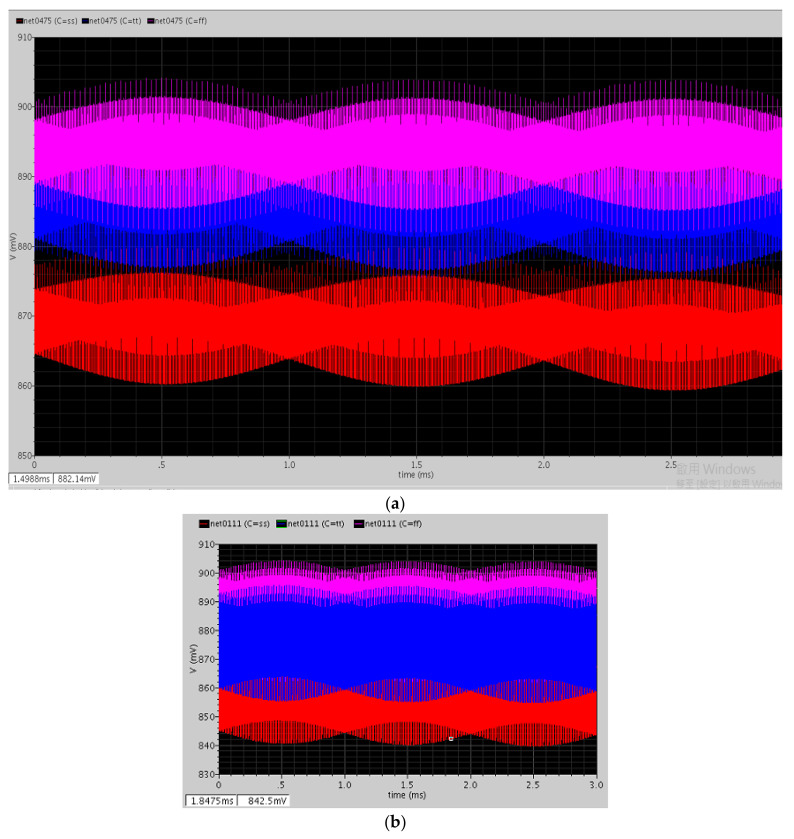
(**a**) After the capacitance to voltage (C/V) circuit; (**b**) after the first demodulation circuit.

We simulated the sensitivity of the MEMS ACC and MAG and the model was then connected with the readout circuit. The test signal for the MAG was a 17.6 kHz sinusoidal wave with the equivalent magnetic field of 500 μT. The frequency of the modulation clock was 400 kHz. Figure 17a is the simulation result of the C/V stage, and the simulation result of the first demodulation is shown in Figure 17b.

**Figure 18:**
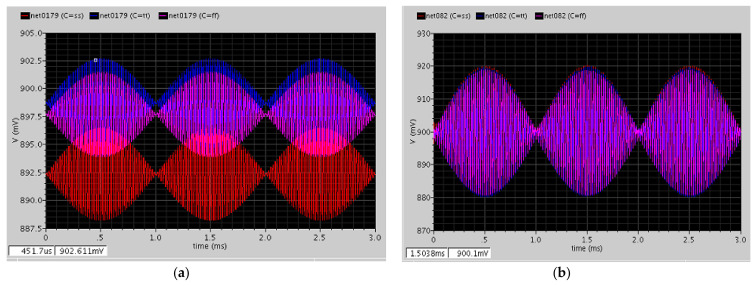
(**a**) After the GM-C filter circuit; (**b**) after the main amplifier circuit.

After the first demodulation, the high-frequency signal was moved to a lower frequency band, and the chopper amplifier had the spike signal. When the first demodulated signal passed through the GM-C filter, the spike signal was reduced as shown in Figure 18a. The DDA was used to lock the common-mode voltage at approximately 0.9 V and is shown in Figure 18b; the output amplitudes of each corner of this stage are similar.

**Figure 19:**
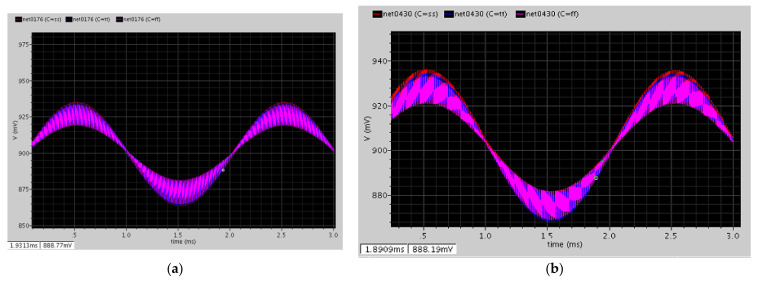
(**a**) After the correlated double-sampling (CDS) demodulation circuit; (**b**) after the buffer circuit.

After the CDS demodulation, the signal returned to the baseband, which was approximately 500 Hz (Figure 19a). Then, the buffer provided enough current to push the capacitor, which was at the nanofarad level, and the gain was one (Figure 19b).

**Figure 20:**
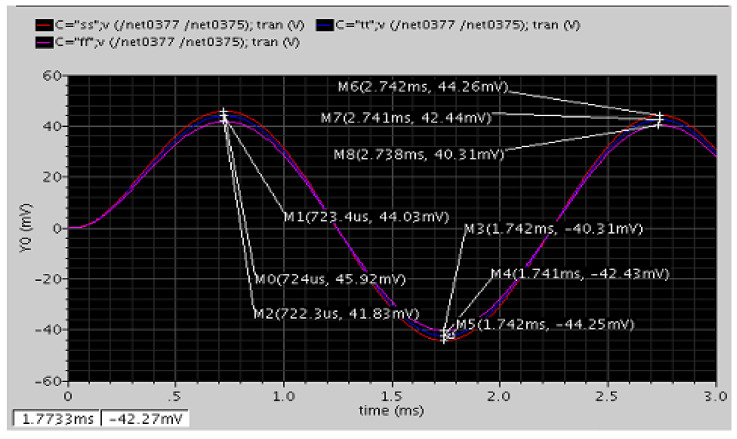
After the two cascading RC filters.

As shown in Figure 20, the two cascading RC filters filter out all the harmonic tones and the high-frequency signals.

Similarly, we can obtain sensitivities of each axis. The sensitivities of the *X*/*Y*/*Z* axes ACC were 88.87/84.87/58.37, 92.68/88.51/60.87, 84.42/80.62/55.45 (mV/g) at different corners, and the sensitivities of the *X*/*Y*/*Z* axes MAG were 9.08/7.1/10.7, 9.46/7.4/11.16, 8.62/6.74/10.17 (mV/uT) at different corners.

**Figure 21:**
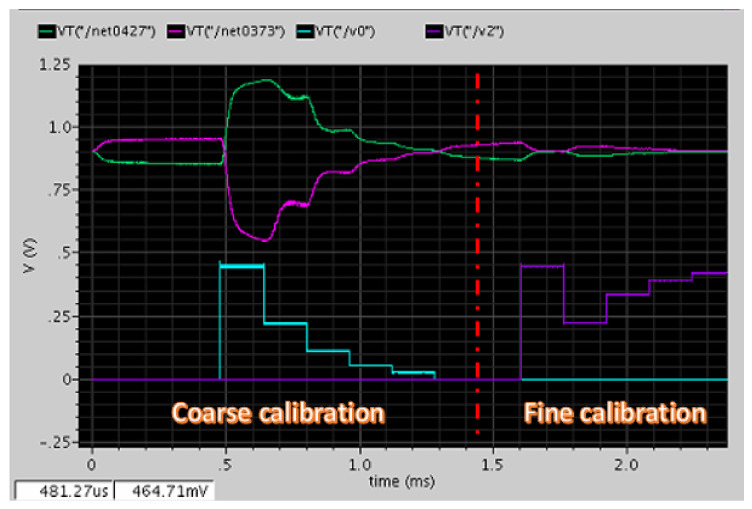
Simulation results of the calibration circuit.

Because the capacitance mismatch in this MEMS process was on average 20%, the calibration circuit was simulated with ±20% capacitance mismatch with zero external signal. Figure 21 shows the simulation result of the calibration circuit. From the illustration, the DC offset in differential sides was saturated at 1.2 V and 0.5 V before calibration. After starting calibration, the SAR logic circuit conducted the 5-bit operation two times with a total of 10 comparisons including the first operation, which was a coarse calibration, and the second operation, which was a fine calibration. Coarse calibration was first done following fine calibration to achieve a 10-bit resolution. After the operation is complete, the DC offset in differential sides was reduced down to approximately 1–10 millivolts (mV). The performance improves even more as long as the $V_{\mathrm{REF}}$ in the DAC circuit is reduced.

Noise contribution is another important specification of the circuit. For the ACC and MAG, the bandwidth of the system was 1000 Hz and 20,000 Hz, respectively. Therefore, by integrating the noise in the bandwidth, the equivalent output referred noise can be obtained. Because this system needs a clock source to drive, periodic stead*Y*-state (PSS) analysis was employed to simulate the period noise of the whole system. The input-referred noises of the readout circuit at 250 Hz were 24.94/30.9/20.84 nV/$\sqrt{\mathrm{Hz}}$ for a TT/SS/FF corner.

Moreover, circuit noise equivalent acceleration (CNEA) and magnetic field (CNEM) can be given by:

(17) $$\mathrm{CNEA}\left(M\right)=\frac{V_{\mathrm{noise}}}{\mathrm{Sensitivity}}$$

where sensitivity is in the unit of farad per acceleration (F/g) and farad per tesla (F/T) for ACC and MAG. The CNEAs of the *X*/*Y*/*Z* axes ACC were 4.83/5.06/7.36, 8.86/6.14/8.92, and 4.11/4.3/6.25 $\mu g/\sqrt{\mathrm{Hz}}$ at different corners. The CNEMs of the *X*/*Y*/*Z* axes MAG were 47.3/60.5/40.1, 57.4/73.4/48.7, and 40.2/51.4/34.1 $\mathrm{nT}/\sqrt{\mathrm{Hz}}$ at different corners.

With BNEA or BNEM and CNEA or CNEM, the system resolution for ACC or MAG can be calculated and is given as in (21), the BNEA is from Formula (7), and BNEM for the *X*/*Y*/*Z* axes are given in Formula (10)–(12). The resolution for the *X*/*Y*/*Z* axes’ ACC and MAG were 5.04/5.26/7.5, 6.03/6.31/9.04, and 4.36/4.54/6.42 μg/√Hz and 64.15/87.46/44.06, 71.92/96.83/52.01, and 59.1/81.43/38.68 nT/√Hz at different corners.

(18) $$\mathrm{Resoultion}=\sqrt{{\mathrm{CNEA}}^{2}+\mathrm{BNEA}{\left(M\right)}^{2}}$$

## 5. Measurement and Discussion

**Figure 22:**
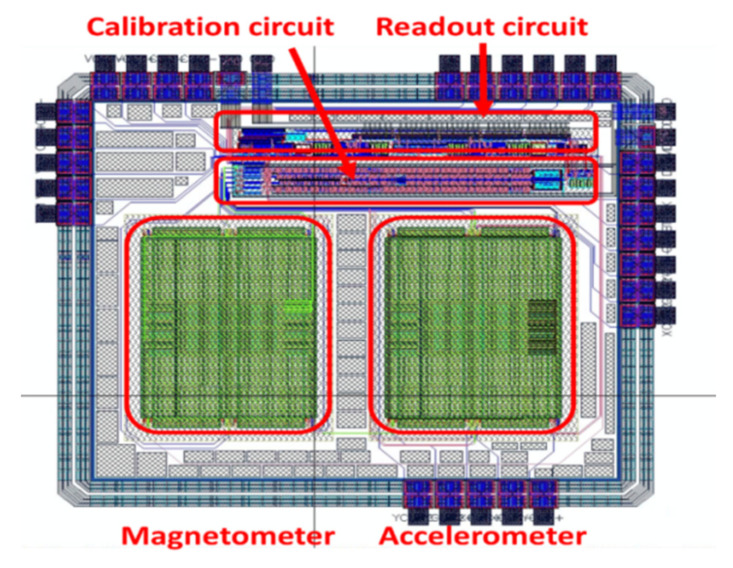
Layout view of the chip.

Figure 22 shows the layout view of the chip. The dimensions of the chip were 1713.96 × 1899.25 µm^2^.

**Figure 23:**
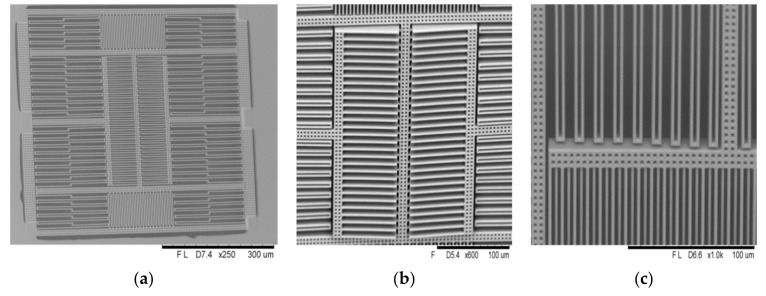
(**a**) Oblique SEM view of the MEMS structure; (**b**) zoomed-in view at the comb-finger; (**c**) zoomed-in view at the beam in the middle.

Figure 23a shows the scanning electron microscope (SEM) view of the MEMS structure in the chip, and the curvature still mainly occurred in the stators on two sides. Figure 23b,c show a zoomed-in view at the comb-finger and beam in the middle and the springs in the corner.

**Figure 24:**
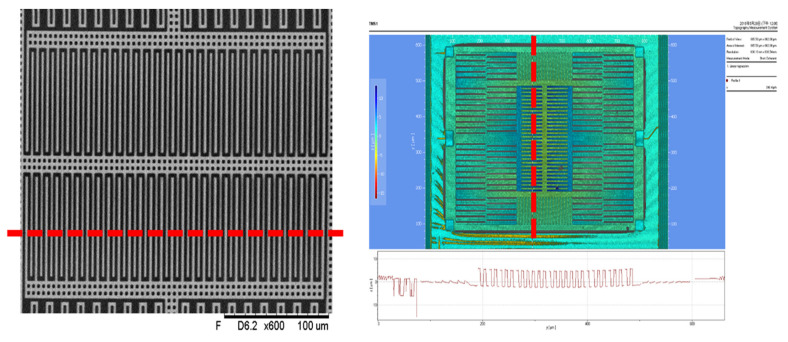
Surface height measurements of the sensing electrodes along the *Y*-axis.

**Figure 25:**
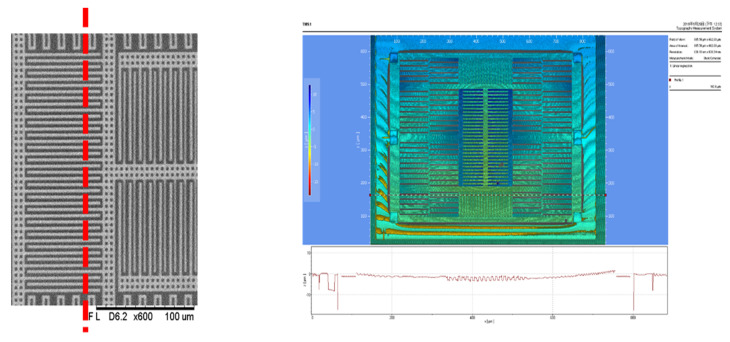
Surface height measurements of the sensing electrodes along the *X*-axis.

Figure 24 and Figure 25 show a top view of the MEMS structure in the white light interferometry (WLI) interface. As shown in Figure 24, for sensing electrodes along the *Y*-axis, the overlapping length of the stator and rotor decreased by 5 µm due to the curvature of the beams. Similarly, in Figure 25, for the sensing electrodes along the *X*-axis, the overlapping length of the stator and rotor decreased by 1.5 µm because of the curvature of the beams and springs. The surface height ($\Delta z_{\max})$ measurement was 7.5 µm in the fingers in the middle, 1.5 µm in the fingers on the sides, 2 µm in the springs, and 2 µm in the beam.

**Figure 26:**
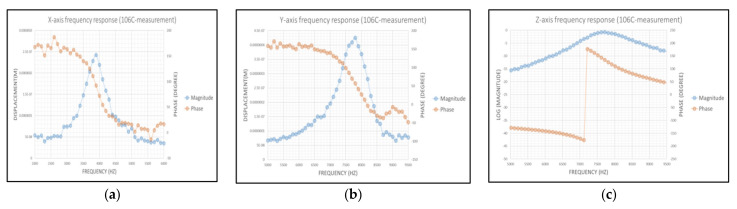
(**a**) Measured bode plot of the MEMS structure along the *X*-axis; (**b**) measured bode plot of the MEMS structure along the *Y*-axis; (**c**) measured bode plot of the MEMS structure along the *Z*-axis.

Figure 26a,b show the frequency response of the motion along the *X*- and *Y*-axis, respectively. The resonance frequency of the *X*-axis and *Y*-axis motion were 3.9 kHz and 7.8 kHz. Moreover, the resonance frequency of the *Z*-axis motion was 7.7 kHz measured by a laser Doppler vibrometer as shown in Figure 26c. In the chip, for resonance frequency, errors between the simulator and measurement were all below 5.28%.

In a printed circuit board (PCB), TLV70218 is used to regulate the supply voltage, OP333 is used as the buffer of reference bias voltage, and INA333 is connected to the output to convert the differential ports to a single end with tunable gain. Switches are used to switch the controlling signal between 0 and 1 without floating the net. The bandwidth of the system was set to be approximately 1000 Hz for the ACC and 20,000 Hz for the MAG by selecting the proper resistor-capacitor (RC) value at the output.

**Figure 27:**
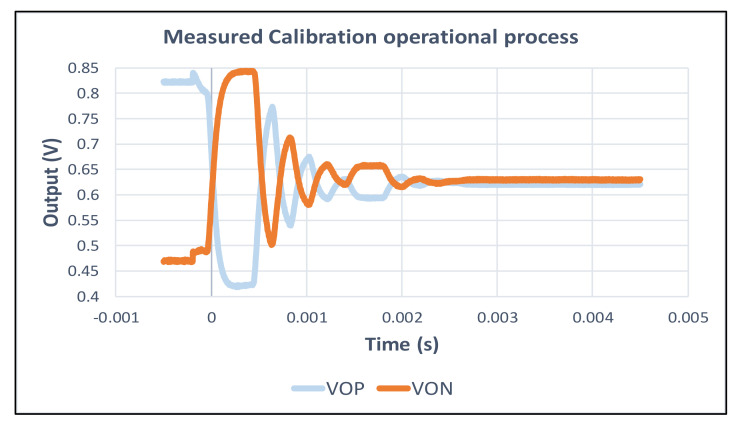
The measurement results of the calibration circuit.

First, the calibration circuit was measured, and the result is shown in Figure 27. There was an offset of 354.7 mV before the calibration. After conducting SAR calibration 10 times, the offset was reduced to 9.72 mV.

**Figure 28:**
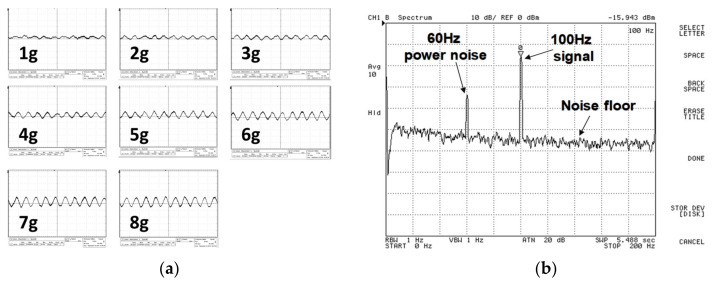
(**a**) Measurement waveform of the accelerometer along the *X*-axis (scale: 6 mV/div); (**b**) measurement spectrum of 4 g acceleration along the *Z*-axis (scale: 10 dB/div).

Then, the PCB board was set on the shaker and the testing accelerating signal was given by 100 Hz with 1–8 g along the *X*-axis. The waveform result is shown in Figure 28a. The sensitivity was approximately 1 mV/g. Noise floor and signal-to-noise ratio (SNR) for 8 g testing signal were 445.372 $\mu V/\sqrt{\mathrm{Hz}}$ and 24.06 dB, respectively.

**Figure 29:**
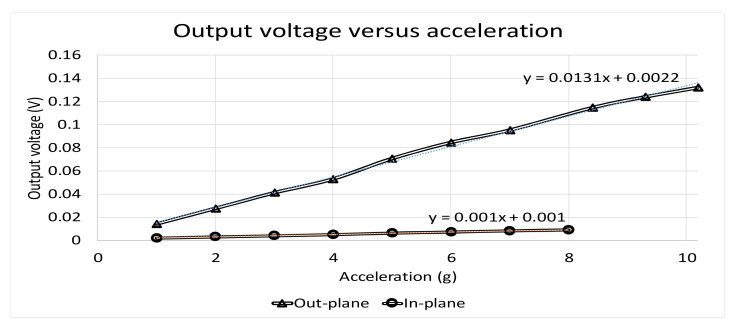
Measurement results of the output voltage versus acceleration.

Similarly, for out-plane motion, the measurement result with 100 Hz and 4 g acceleration in the network analyzer is shown in Figure 28b. Noise floor and SNR for the 4 g testing signal were 445.372$\mu V/\sqrt{\mathrm{Hz}}$ and 40.124 dB, noise floor and SNR for the 8 g testing signal were 445.372 $\mu V/\sqrt{\mathrm{Hz}}$ and 46.4 dB, respectively. Figure 29 shows the relationship between input acceleration and output voltage, the sensitivity was approximately 13.1 mV/g for out-plane motion.

**Figure 30:**
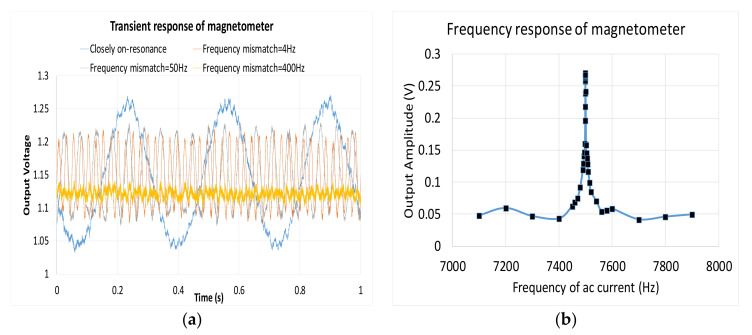
Measured (**a**) transient and (**b**) frequency response of magnetometer.

Moreover, the measurement results of the MAG are shown. First, we can check the resonance frequency of the MEMS structure by supplying an A/C Lorentz current with different frequencies. For out-plane motion, Figure 30a measured the MAG transient and the frequency response of the output voltage under different frequencies of the MAG (Figure 30b). As the frequency of the A/C current approached the mechanical resonance frequency, the amplitude of the output increased. Table 2 presents a comparison of both the MAG and ACC.

## 6. Conclusions

In this paper, we studied microelectromechanical systems (MEMS) with multi-functions including a three-axis MAG, a three-axis ACC, and backend circuitry. The sensitivity and resolution for three-axis ACC were 58.37–88.87 uV/ug and 5.043–7.5 ng/√Hz. The sensitivity and resolution for three-axis MAG were 7.1–10.7 uV/uT and 44.06–87.46 nT/√Hz. The resolution was limited by CNEM and BNEM.

Moreover, two solutions for curvature have been presented: The first solution is that the readout circuit uses noise reduction technology, the frequency division multiplexing method, and the time-division multiplexing method. The second solution is the implemented the capacitance calibration circuit. After conducting SAR calibration 10 times, the offset was reduced to 9.72 mV and reducing the curvature that causes problems such as the reduction sensitivity and the enlargement in offset. However, there are some drawbacks, such as the curvature still being too large and the dynamic range of the MAG too small. These two phenomena will make the design of the circuit more difficult. From the measurement results, we observed that a large curvature usually occurs in the long structures such as the beams, fingers, and springs. Therefore, if we can sacrifice sensitivity to compensate for the reduction in curvature, and the Lorentz current with an oscillator of the MAG to enhance a large dynamic range of reading, difficulties in the readout circuit might be solved.

## Figures and Tables

**Table 1:** Dimensions of the MEMS magnetometer.

Position	Value	Position	Value
Proof mass length (μm)	299	Stator fingers (D) (μm)	75.4
Proof mass width (μm)	13	Rotor fingers (E) (μm)	75.4
Finger width (A) (μm)	2.6	Spring width (F) (μm)	2.6
Finger gap width (B) (μm)	2.6	Spring width parallel (G) (μm)	2.6
Finger overlap length (C) (μm)	72.8	Spring width perpendicular (H) (μm)	7.8
	-	Spring length (I) (μm)	221

**Table 2:** Summary of the specifications of the MEMS magnetometer and accelerometer.

Specification	[7]	[1]	[2]	[8]	[5]	[4]	[3]	This Paper	This Paper
Type	MAG	MAG	MAG	MAG	MAG	MAG	ACC	ACC	MAG
Axis	Three	*Y*-	Three	*X*-/Z-	Z-	Three	Three	Three	Three
Sensitivity (uV/uT(g))	250	210000	24.2 pF/T	3.2	150	1.51	1.3 fF/g	58.37–88.87	7.1–10.7
Resolution (nT(g)/√Hz)	4	0.00276	17	344	2080	121.6	75	5.043–7.5	44.06–87.46
Fs									
Mag drive Current (mA)	1.08	7.245	5.8	4.5	0.25	4.02	-	-	2

## Data Availability

Relevant information can be obtained through the laboratory.
